# Hospital Sunday in London

**Published:** 1895-06-15

**Authors:** 


### The Hospital

An Institutional Journal of

The Medical Sciences and Hospital Administration, with

AN EXTRA NURSING SUPPLEMENT.

Vol. XVIII.—No. 455.] [June 15, 1895.

### Hospital Sunday in London.

It is a prodigious task to provide more than a third of the whole population of the metropolis with free medical treatment, physic, surgical appliances, and nursing from year to year. And yet, though so prodigious, it is always done, and always well done. It has taken the conductors of this journal almost ten years of unceasing effort to demonstrate the significance of the hospital life and work of London, and now, at the close of that period, the feeling is that those who know most of it, and realise it most fully, are most conscious of their failure to grasp in any adequate sense the full significance of it all: its significance on the one hand to those who give, and on the other to those who receive.

Exultation and depression take their places by turns in the mind of the thoughtful student of hospital life and work. That so much should have been done for so many years past; that to our fathers and ourselves is due the monumental honour of having achieved it; that an object lesson in voluntary goodwill, as opposed to compulsory and rate-paid service, should have been given to all the world, and admitted by all the world to have no parallel in its greatness—these facts make the living hospital worker proud and exultant. But there is another side to the shield. How is it all to be maintained in the future? How is even the very next year to be provided for? What the hospital worker sees, or thinks he sees, is a vast lethargic population, who, though knowing the nature of the work to be done, and the prodigious task of doing it, yet leave to a mere handful of benevolent and energetic persons, not the honour and glory, for that all London shares, but the strenuous and gigantic labour of carrying a burden which year by year grows heavier and more important to the inhabitants of London.

We do not believe that hospital supporters and hospital workers will break down in the presence of the task they have undertaken. They will carry it on and make it greater. It is a part of the moral and social life of our race and our generation. It is as much a part of our duty as the maintenance of the national honour, and the extension of the national influence in every part of the globe. It is something committed to our race and generation by the highest authority. But, in spite of all this, nothing can save hospital life and work from collapse except an unyielding resolution on the part of those who take it up to strengthen and extend it in every part of the metropolis and the country and to bring under its influence and to its aid every right-minded man, woman, and child who has power to influence others or capacity for personal help. If hospital life and work are to be continued at their present high and noble level, it can only be by the gradual conversion of the whole community into hospital supporters and workers.

What is the immediate task before us? It is the making of a Hospital Sunday collection, says the man of routine; and since such collections have been made for nearly a quarter of a century, what need is there for special fuss at the present moment? There is no need for special fuss; there is no need for "fuss" at all; fuss will not do what is wanted. The truth is that the coach of Hospital Sunday has got into a deep rut; she has been in that rut for several years; and it is absolutely imperative that she be got out of the rut. It is not the first time the gallant coach has been brought to a standstill in this way. A good many years ago she fell into a similar but even narrower rut, the rut of twenty thousand pounds; and there she stuck almost immovable for several years. It was then perceived that such a stopping of the coach and a blocking of the roadway were disgraceful; and by a united effort the coach was lifted out of the rut, and bowled merrily along the road until forty, and even forty-five thousand pounds were reached. Then a stoppage. The coach is once more in the rut, the rut of forty thousand pounds. But we can no more stay permanently at forty thousand pounds than could our fathers at twenty thousand. The hour has come for a new united effort; the hour, and, we hope, the men and the women. Not forty, but seventy thousand pounds is the amount to be raised this year. The leap from twenty to forty thousand pounds was a doubling of former work at a single stroke. We do not ask for the Sunday collection to be doubled this year; though if it should be the hospitals can make use of it all. But we do ask, and with all the energy of convinced necessity, that seventy thousand and no less shall be the next point which, by united effort, the hospital coach shall reach.

In this article we make no reference to detailed figures. The figures, presented in a variety of novel and convincing ways, tell their own story in the pages of our special supplement. But we ask most insistently that those figures shall be studied. One figure we may present, the figure of 1,717,187, a prodigious figure. This represents the incredible number of the men, women, and children relieved in our London hospitals last year. One more large figure we may also give, the figure of £790,229. This represents the cost of the work. Yet one more, and the bird's-eye view of the figures is complete: it is the figure of £766,245, and it represents the income from all sources of our voluntary medical charities. There is thus a deficit of close upon £25,000 to be reckoned with. The Hospital Sunday Fund could, if it would, by a single stroke, by the united effort of lifting the coach out of the rut, at once and permanently put an end to this deficit. If any person who has hitherto given a guinea will give a guinea and a-half on Sunday next, and every one who has not given more than a shilling will give two shillings, and those who have given nothing heretofore will now give their share, the thing will be done. We have purposely presented a simple story to our readers, and told them of a simple work to be done next Sunday. But that work, though so simple and easy to each individual who is asked to take his share of it, would mean the secure establishment of the resources of our hospitals on a firm and permanent basis, the saving of hundreds of lives, and the bringing of sunshine and happiness into thousands of metropolitan homes. Let us but make a strong and united effort and the thing is done.

